# Association of frailty with health service utilisation and health care expenditure in sub-Saharan Africa: evidence from Côte d’Ivoire

**DOI:** 10.1186/s12877-021-02377-6

**Published:** 2021-07-30

**Authors:** Rachel C. Ambagtsheer, Richard K. Moussa

**Affiliations:** 1 National Health and Medical Research Council Centre of Research Excellence in Trans-Disciplinary Frailty Research to Achieve Healthy Ageing, Adelaide, Australia; 2 Torrens University Australia, GPO Box 2025, Adelaide, SA 5000 Australia; 3 Ecole Nationale Supérieure de Statistique et d’Economie Appliquée, Abidjan, Côte d’Ivoire; 4 Institut National de la Statistique, Abidjan, Côte d’Ivoire

**Keywords:** (MESH): frailty, Aged, Aged, 80 and over, Health services, Africa south of the Sahara

## Abstract

**Background:**

Frailty, a syndrome resulting in heightened risk of negative outcomes for older adults, is increasing across the globe. However, little is known about the health service impacts of frailty in low-income countries (LICs), and in particular, sub-Saharan Africa (SSA). This study explores the relationship between frailty and health service 1) utilisation and 2) expenditure within Côte d’Ivoire.

**Methods:**

Participants aged 50 years and over participated in the Living Condition, Health and Resilience among the Elderly study. Frailty was assessed using a 30-item Frailty Index (FI). The association between frailty and self-reported health service utilisation was analysed for general practitioners (GPs), specialists, overnight hospitalisations, traditional practitioners and self-medication. Expenditure over the previous month included consulting, medications, hospitalisations and total expenditure.

**Results:**

Among participants [*n* = 860, mean age (SD) = 61.8 (9.7) years, 42.9% female], 60.0% were frail, 22.8% pre-frail and 17.2% robust. The mean (SD) FI was 0.28 (0.17). Increased health service utilisation was associated with frailty for GP attendance, traditional practitioners and self-medication but not specialists or overnight hospitalisation. Pre-frailty and frailty were associated with increased total health service expenditure, with frailty also associated with aggregate consulting costs and medications.

**Conclusions:**

Although frailty is associated with health service utilisation and expenditure in a variety of contexts, the study results suggest that such impacts may vary across the globe. The experience of frailty in LICs is likely to differ from that experienced elsewhere due to cultural traditions, attitudes to the health system, and accessibility, with more research needed.

**Supplementary Information:**

The online version contains supplementary material available at 10.1186/s12877-021-02377-6.

## Background

Over the decades between 2020 and 2050, world populations will experience unprecedented growth in population ageing, and with it, a rapid increase in the proportion and number of older adults living with frailty [1]. Frailty is a relatively common clinical syndrome of declining physiological reserve across multiple systems that results in an elevated risk of adverse outcomes for older adults including falls, increased hospitalisation and early mortality [2, 3]. Within high income countries (HICs) there is an increasing emphasis on early identification and treatment of frailty given emerging evidence that frailty is a dynamic [4, 5] and potentially treatable condition [6]. However, despite a faster progression of ageing in low-income and middle-income countries (LMICs) than in HICs [7, 8], there is a dearth of information about the impacts of frailty within these regions [7, 9, 10]. In sub-Saharan Africa (SSA), where the older population is projected to triple between now and 2050 [11], frailty analyses are very scarce, in part due to a lack of high quality, comprehensive data sets addressing the needs of older adults [12].

Several previous studies conducted within HICs have observed a positive relationship between frailty and health service expenditure and usage respectively. Frailty, particularly in its advanced stages, has been associated with increased health care costs in a number of previous studies [13–16]. Frailty has also been demonstrated to increase usage of hospital and community care services, specialist services and emergency department presentations, along with visits to a range of health service providers, although the evidence for increased general practitioner (GP) attendance is mixed [14, 17–20]. However, there are a number of reasons why this association might differ within the SSA context. Firstly, care provision for older adults is largely provided by families, rather than formal health systems, especially with regard to long-term care [11]. Secondly, the prevalence of frailty within LMICs may have a different profile to that within HICs, although the supporting evidence is currently inconsistent. Despite one systematic review finding higher frailty prevalence in LMICs than HICs [7], a large multi-country study reported the opposite case [21]. Potential reasons that have been advanced for this discrepancy could include lower life expectancy in LMICs, along with under-reporting of medical conditions and relatively lower access to care [7]. Thirdly, the added complication of HIV-infection, which has been associated with increased frailty and premature ageing within one SSA study [22], might be expected to have a compounding effect on both health service costs and usage. It is unclear what the overall effect of these differences might be with respect to the overall impact on the relationship between frailty, health service costs and health service utilisation.

Consequently, the aim of the present study was to explore the relationship between frailty and 1) health service utilisation and 2) health service expenditure among older adults living within an LMIC (sub-Saharan African) context. A related objective was to determine the associations of frailty with key socio-demographic characteristics within this cohort. We hypothesised that there would be a positive relationship between both frailty and health service utilisation and frailty and health service expenditure respectively.

## Methods

### Study setting

The Living Condition, Health and Resilience among the Elderly study (Enquête sur les conditions de vie, la Santé et la Résilence des personnes âgées) was conducted by the Ecole Nationale Supérieure de Statistique et d’Economie Appliquée (ENSEA) and funded by the African Development Bank (AfDB). It was conducted within two départements (sub-regions) of Côte d’Ivoire: Dabou in the South, and Toumodi, in the centre (Table 1). The study was approved by the Comité Consultatif National de Bioéthique de la République de Côte d’Ivoire (National Ethical Committee of Côte d’Ivoire).

**Table 1:** Study area statistics by sub-region (Source: 2014 Census)

Statistics	Dabou	Toumodi
Total Population	148,874	127,825
Total Female	72,883 (49.0%)	63,104 (49.4%)
Total Ivorians	113,978 (76.6%)	115,623 (90.5%)
Average household size	4.8	5.7
Total Muslims	47,078 (31.6%)	23,741 (18.6%)
Total Christians	92,244 (62.0%)	60,496 (47.3%)
Population aged 0–34 years	112,376 (75.5%)	95,319 (74.6%)
Population aged 65 years and above	4437 (3%)	5999 (4.7%)
% Illiterate (among 15 years and above)	40.0%	58.0%
Urbanization rate	49.3%	46.5%
Density (inhabitants per sq. km)	66	45

### Participants

Participants were selected via a multi-stage cluster sampling process. In the initial step, each sub-region was divided into two clusters (urban and rural). In rural clusters, the proportion of households surveyed was 100.0% for villages of < 150 inhabitants, 50% for 150 to 400 inhabitants, 33% for > 400 to 700 inhabitants and 20% for villages with > 700 inhabitants. In the urban cluster (the town itself), 5% of households were surveyed. Within each selected household, one adult (aged ≥18 years) was randomly selected to be interviewed. The sample obtained for each “département” was representative of its underlying population structure. Informed oral consent was obtained from all participants. With the Dabou region, 2775 households (2775 household members) were sampled, of which 2537 were interviewed (a response rate of 91.4%). Participants aged at least 50 years within this region numbered 366. Within the Toumodi region, 1975 households and 1975 household members were sampled, with 1918 interviewed (a response rate of 91.1%). Participants aged at least 50 years within this region numbered 494.

### Data collection

Data collection was conducted during two time periods over 2 years: with the first wave undertaken in May 2017 in Dabou and the second in July 2018 in Toumodi. Each wave corresponded to data collection within a single département. Face to face interviews were conducted by ENSEA students in French under the supervision of their assistant professors. On average, each interview took approximately 35 min to complete. Data were collected using tablets loaded with CSPro software and subsequently imported into Stata v.16.0.

For the purpose of our study, we extract from the overall dataset the subpopulation of those aged 50 or more.

### Frailty measure

Frailty was measured using a 30-item self-reported Frailty Index (FI) constructed according to the deficit accumulation method [23, 24]. The FI incorporated multiple domains, including mobility restriction, chronic diseases, activity limitations, mental health, pain and cognition. As per previous research, a threshold of > 0.21 was applied to denote frailty, with pre-frailty defined as the range > 0.1 to ≤0.21 and non-frailty as ≤0.1 [25].

### Control variables

A range of socio-demographic variables were used as control variables in the statistical analyses, including age group, sex, marital status, health insurance, area of residence, location, employment status and education level. Selection of variables was based on prior research along with consideration of potential factors likely to influence the outcome measures (e.g. health insurance status). Age was collected as a continuous variable (measured in years), and five-year age groups were created on this basis. Two gender categories were created (1 if male, 0 if female). Marital status was collected within four categories: whether the individual has never been married, currently living with a partner, divorced or widowed. Educational level categories were as follows: university, high school, primary school, or never schooled. The four employment categories were: employed, unemployed, home-worker or out of the labor market. Two health insurance categories were created (1 if the individual had private, public or other type of health insurance, and 0 if no health insurance). Two location areas were considered: the regions of Dabou and Toumodi, while areas were defined as urban or rural according to national definitions.

### Outcome measures

Our two primary outcome measures were 1) health service utilisation and 2) health service expenditure. Health service utilisation was measured by asking participants if they had used any of the following services over the past 12 months: hospital (overnight stay), general practitioner (GP), specialist doctor, self-medication or traditional practitioner. For hospitalisations, GPs and specialist doctors, participants were asked how many times they had used the service over the past 12 months. In terms of health service expenditure, participants were asked what their total health expenditure, their consulting costs (GPs and specialists), medication costs and hospital expenses were over the past month.

### Data analysis

All data analysis was conducted in Stata v.16.0. Descriptive statistics were reported using mean and standard deviation for continuous variables, and count and proportion for categorical variables. To estimate the strength of the relationship between the health service utilisation variables and frailty, logistic regression was used when the outcome variables were binary. For count outcomes, negative binomial regression was used after testing for overdispersion in poisson models. To estimate the strength of the relationship between the health service expenditures variables and the frailty category, as a robustness analysis to the standard Tobit regression model, two-part models with a logistic model for the first part and a gamma regression model with log link for the second part were estimated [26], since the cost variables included zero values. Six models were applied which controlled for various combinations of the control variables, from Model 1 (no control variable included) up to Model 6 (all control variables included). As for robustness analysis, the following thresholds: (i) 0.25, (ii) 0.31, (iii) 0.35, and (iv) 0.41 were used to denote frailty. For each of these thresholds, we estimated Model 6 for each of the outcomes. Results of these estimates are included in the Supplementary Data.

## Results

### Participant characteristics

Participant characteristics by frailty status and key socio-demographic variables are shown in Table 2. Of 860 total participants, the mean age was 61.8 years (9.7), with 42.9% female. The majority (60.0%) of participants were frail as determined by the Frailty Index, with a further 22.8% pre-frail and 17.2% robust. Frail participants were more likely to be older, have no health insurance, be living in rural areas, be living in the South, have lower educational attainment, be a house worker or out of the labour force and to be widowed than non-frail participants. The mean (SD) FI was 0.28 (0.17), with a range from 0.0 to 0.73. An overall trend was observed for the prevalence of both pre-frailty and frailty to increase with age.

**Table 2:** Participant characteristics by frailty status

	Robust (< 0.10)		Pre-Frail (0.10–0.21)			Frail (> 0.21)		
	*n* = 148		*n* = 196			*n* = 516		
	n.	%	n	%	*p* value†	n	%	*p* value ‡
Sex								
Male	108	22.0	129	26.3	0.117	254	51.7	< 0.001
Female	40	10.8	67	18.2	0.005	262	71.0	< 0.001
Health insurance								
Yes	20	23.3	30	34.9	0.093	36	41.9	0.009
No	128	16.5	166	21.4	0.014	480	62.0	< 0.001
Age groups								
50–54	42	19.8	55	26.0	0.133	115	54.2	< 0.001
55–59	42	20.9	56	27.9	0.104	103	51.2	< 0.001
60–64	35	20.7	33	19.5	0.786	101	59.8	< 0.001
65–69	15	11.4	27	20.4	0.044	90	68.2	< 0.001
70–74	8	13.8	14	24.1	0.155	36	62.1	< 0.001
75–79	2	5.3	5	13.1	0.234	31	81.6	< 0.001
80 +	4	8.0	6	12.0	0.505	40	80.0	< 0.001
Area of residence								
Rural	104	16.5	140	22.2	0.010	387	61.3	< 0.001
Urban	44	19.2	56	24.5	0.175	129	56.3	< 0.001
Location								
South	41	11.2	74	20.2	< 0.001	251	68.6	< 0.001
Centre	107	21.7	122	24.7	0.258	265	53.6	< 0.001
Education level								
Not schooled	42	12.5	56	16.8	0.126	237	70.7	< 0.001
Primary	32	14.7	51	23.5	0.020	134	61.8	< 0.001
High school	52	21.0	69	27.8	0.076	127	51.2	< 0.001
University	22	36.7	20	33.3	0.702	18	30.0	0.439
Employment status								
Employed	96	20.8	114	24.8	0.158	251	54.4	< 0.001
Unemployed	3	17.6	3	17.7	1.000	11	64.7	0.005
House worker	18	12.8	29	20.5	0.079	94	66.7	< 0.001
Out of labour force	31	12.9	50	20.7	0.021	160	66.4	< 0.001
Marital status								
Not married	21	22.1	25	26.3	0.498	49	51.6	< 0.001
In couple**	106	19.3	134	24.5	0.041	308	56.2	< 0.001
Divorced	8	19.0	8	19.1	1.000	26	61.9	< 0.001
Widowed	13	7.4	29	16.6	0.009	133	76.0	< 0.001

†Pre-Frail vs. Robust; ‡ Frail vs. Robust; ** means living with a partner, with or without formal marriage

### Health service utilisation

In terms of health service utilisation, the prevalence ratio (PR) for consulting a GP, consulting a traditional practitioner or self-medicating within the previous 12 months was consistently higher for frail than for non-frail older adults across all models analysed (Table 3). For example, under Model 6, which controlled for all covariates, the PR for the frail sub-group in comparison with the non-frail sub-group was 2.1 (95% CI: 1.4–2.7, *p* = 0.002) for GP attendance, 2.8 (95% CI: 1.1–4.6, *p* = 0.040) for consulting a traditional practitioner and 1.8 (95% CI: 1.3–2.3, *p* = 0.004) for self-medication.

**Table 3:** Cross-sectional associations for frailty and health service utilisation: health service usage in last 12 months

	Model 1: unadjusted PR (95% CI) [*p*-val.]	Model 2: adjusted PR (95% CI) [*p*-val.]	Model 3: adjusted PR (95% CI) [*p*-val.]	Model 4: adjusted PR (95% CI) [*p*-val.]	Model 5: adjusted PR (95% CI) [*p*-val.]	Model 6: adjusted PR (95% CI) [*p*-val.]
Admitted to hospital overnight in last 12 months						
Pre-frail	4.5 (− 0.9–10.0) [0.205]	4.5 (− 0.9–9.8) [0.205]	4.5 (− 0.9–10.0) [0.205]	4.6 (− 1.0–10.2) [0.203]	4.3 (−0.9–9.6) [0.213]	4.3 (− 0.9–9.4) [0.215]
Frail	7.2 (− 1.0–15.3) [0.139]	7.1 (− 1.1–15.2) [0.145]	7.3 (− 1.1–15.6) [0.141]	7.5 (− 1.1–16.2) [0.138]	6.4 (−1.0–13.8) [0.155]	6.4 (− 1.1–14.0) [0.159]
General practitioner in last 12 months						
Pre-frail	1.3 (0.8–1.7) [0.283]	1.2 (0.8–1.7) [0.325]	1.2 (0.8–1.7) [0.306]	1.2 (0.8–1.7) [0.307]	1.3 (0.8–1.7) [0.263]	1.2 (0.8–1.6) [0.380]
Frail	2.1 (1.4–2.7) [0.001]	2.0 (1.4–2.7) [0.002]	2.2 (1.5–2.8) [0.001]	2.0 (1.4–2.7) [0.002]	2.2 (1.5–2.8) [0.001]	2.1 (1.4–2.7) [0.002]
Consulted a specialist doctor in last 12 months						
Pre-frail	2.0 (0.6–3.3) [0.168]	2.0 (0.6–3.5) [0.151]	1.9 (0.6–3.2) [0.167]	2.0 (0.6–3.4) [0.167]	2.1 (0.6–3.5) [0.149]	2.1 (0.6–3.5) [0.144]
Frail	2.0 (0.7–3.2) [0.135]	2.1 (0.7–3.4) [0.113]	2.2 (0.9–3.6) [0.073]	2.0 (0.7–3.4) [0.131]	2.3 (0.8–3.7) [0.085]	2.5 (0.9–4.1) [0.067]
Self-medication in last 12 months						
Pre-frail	1.4 (1.0–1.9) [0.062]	1.4 (1.0–1.9) [0.0646]	1.4 (1.0–1.9) [0.057]	1.5 (1.0–1.9) [0.054]	1.4 (1.0–1.9) [0.058]	1.5 (1.0–1.9) [0.052]
Frail	1.7 (1.2–2.2) [0.004]	1.7 (1.2–2.2) [0.0035]	1.7 (1.2–2.1) [0.006]	1.7 (1.3–2.2) [0.003]	1.7 (1.2–2.2) [0.004]	1.8 (1.3–2.3) [0.004]
Traditional practitioner in last 12 months						
Pre-frail	1.9 (0.6–3.1) [0.177]	1.8 (0.6–3.1) [0.179]	1.9 (0.6–3.1) [0.170]	1.9 (0.6–3.1) [0.171]	1.8 (0.6–3.1) [0.179]	1.9 (0.6–3.1) [0.166]
Frail	2.8 (1.1–4.5) [0.033]	2.8 (1.1–4.5) [0.037]	2.7 (1.1–4.3) [0.037]	2.8 (1.2–4.5) [0.031]	2.7 (1.1–4.4) [0.040]	2.8 (1.1–4.6) [0.040]

Reference group: robust (non-frail) *PR* prevalence ratio; *CI* confident interval; *p-val. p*-value of the test of H_0_: PR = 1; *ref*. reference category; model 1:unadjusted; model 2: adjusted by sex, age groups, and marital status; model 3: adjusted by health insurance; model 4: adjusted by area of residence and location; model 5: adjusted by employment status and education level; model 6: adjusted by sex, age groups, marital status, health insurance, area of residence, location, employment status and education level. The number of observations in all estimations is *n* = 860

In contrast, being frail was not associated with a higher PR for being hospitalised overnight nor consulting a specialist doctor within the same period. Further, no strong evidence for an association of pre-frailty with increased health service utilisation for any type of health service provision was observed, although a non-significant gradient effect was observed with increasing frailty.

When the number of consultations within the last 12 months was analysed (Table 4), frailty was associated only with the number of consultations with a GP across all models (Model 6 results: PR = 2.4, 95% CI: 1.2–3.5, *p* = 0.019) and not with overnight hospital admission or specialist doctor attendance. Pre-frailty was not associated with an increased number of consultations of any kind.

**Table 4:** Cross-sectional associations for frailty and health service utilisation: number of consultations in last 12 months

	Model 1: unadjusted PR (95% CI) [p-val.]	Model 2: adjusted PR (95% CI) [*p*-val.]	Model 3: adjusted PR (95% CI) [*p*-val.]	Model 4: adjusted PR (95% CI) [*p*-val.]	Model 5: adjusted PR (95% CI) [*p*-val.]	Model 6: adjusted PR (95% CI) [*p*-val.]
Number of times being admitted to hospital overnight in last 12 months (with zeros)						
Pre-frail	2.2 (− 1.1–5.4) [0.472]	2.8 (− 0.5–6.0) [0.284]	2.2 (− 1.0–5.4) [0.475]	2.1 (−1.0–5.2) [0.484]	2.4 (−0.7–5.6) [0.372]	2.9 (− 0.1–5.9) [0.208]
Frail	4.0 (−1.7–9.6) [0.299]	4.5 (−0.7–9.7) [0.182]	4.0 (−1.7–9.7) [0.303]	4.3 (− 1.8–10.4) [0.285]	3.7 (−1.2–8.7) [0.273]	4.9 (−0.0–9.8) [0.122]
Number of times consulting a general practitioner in last 12 months (with zeros)						
Pre-frail	1.8 (0.6–2.9) [0.183]	1.3 (0.6–2.0) [0.427]	1.8 (0.6–3.0) [0.171]	1.7 (0.7–2.6) [0.197]	1.7 (0.6–2.7) [0.205]	1.3 (0.6–2.1) [0.380]
Frail	3.1 (1.6–4.6) [0.005]	2.3 (1.2–3.4) [0.020]	3.4 (1.8–5.1) [0.004]	2.9 (1.6–4.3) [0.005]	3.0 (1.5–4.5) [0.010]	2.4 (1.2–3.5) [0.019]
Number of times consulting a specialist doctor in last 12 months (with zeros)						
Pre-frail	6.2 (0.7–11.8) [0.063]	7.0 (1.3–12.7) [0.041]	7.4 (1.2–13.6) [0.045]	6.7 (0.9–12.4) [0.054]	5.3 (0.8–9.8) [0.062]	6.0 (0.9–11.1) [0.056]
Frail	3.9 (1.1–6.7) [0.042]	4.8 (1.4–8.3) [0.030]	6.3 (1.5–11.0) [0.029]	3.8 (1.1–6.5) [0.039]	5.3 (0.9–9.7) [0.054]	7.2 (0.6–13.7) [0.064]

Reference group: robust (non-frail) *PR* prevalence ratio; *CI* confident interval; *p-val*. *p*-value of the test of H0: PR = 1; *ref*. reference category; model 1: unadjusted; model 2: adjusted by sex, age groups, and marital status; model 3: adjusted by health insurance; model 4: adjusted by area of residence and location; model 5: adjusted by employment status and education level; model 6: adjusted by sex, age groups, marital status, health insurance, area of residence, location, employment status and education level. The number of observations in all estimations is *n* = 860

### Health service expenditure

The results of our two-part models (Tables 5 and 6) are consistent with the literature on this topic [27]. From our unadjusted as well as our adjusted models, we find that being frail or pre-frail increases the odds of incurring a health care expenditure compared with being non-frail. The occurrence of a health care expenditure is 1.75 to 1.93 times higher for pre-frail than non-frail while it is 2.66 to 3.71 times higher for frail in comparison with non-frail. When we consider the types of expenditure, we find that the occurrence of consulting expenditures, medication expenditures and hospitalization expenditures are respectively 1.57 to 1.60, 1.52 to 1.63, and 1.49 to 1.63 times higher for pre-frail in comparison with non-frail. The frail individuals have a risk of occurrence of consulting expenditures, medication expenditures and hospitalization expenditures that are respectively 2.20 to 2.51, 2.46 to 3.22, and 2.44 to 3.22 times higher than the non-frail.

**Table 5:** Two-part models on cost variables

	Model 1: unadjusted		Model 2: adjusted		Model 3: adjusted	
	Logit OR (95% CI) [*p*-val.]	ME (95% CI) [*p*-val.]	Logit OR (95% CI) [*p*-val.]	ME (95% CI) [*p*-val.]	Logit OR (95% CI) [*p*-val.]	ME (95% CI) [*p*-val.]
In the past month, how much did you spend on consulting costs?						
Pre-frail	1.58 (0.94–2.66) [0.080]	− 0.39 (− 8.08–7.30) [0.921]	1.62 (0.96–2.75) [0.070]	1.24 (−4.24–6.71) [0.658]	1.58 (0.93–2.66) [0.090]	−0.36 (− 7.86–7.14) [0.925]
Frail	2.25 (1.43–3.53) [< 0.001]	4.60 (− 3.42–12.62) [0.261]	2.32 (1.45–3.67) [< 0.001]	5.50 (0.03–10.96) [0.049]	2.51 (1.58–3.97) [< 0.001]	5.13 (− 3.65–13.91) [0.252]
In the past month, how much did you spend on medications (drugs)?						
Pre-frail	1.52 (0.95–2.41) [0.078]	−0.77 (− 6.45–4.90) [0.789]	1.55 (0.97–2.48) [0.064]	− 1.01 (− 6.60–4.58) [0.724]	1.51 (0.94–2.44) [0.089]	− 0.86 (− 6.36–4.63) [0.758]
Frail	2.46 (1.67–3.67) [< 0.001]	9.87 (3.54–16.20) [0.002]	2.64 (1.73–3.97) [< 0.001]	8.49 (2.40–14.57) [0.006]	2.8 (1.86–4.22) [< 0.001]	10.35 (4.06–16.64) [0.001]
In the past month, how much did you spend on hospitalisation expenses?						
Pre-frail	1.54 (0.45–5.21) [0.493]	0.88 (− 1.02–2.77) [0.365]	1.49 (0.44–5.05) [0.525]	0.84 (− 0.55–2.24) [0.237]	1.51 (0.44–5.16) [0.506]	0.74 (− 0.82–2.30) [0.351]
Frail	2.69 (0.94–7.69) [0.064]	5.61 [1.42–9.80) [0.009]	2.64 (0.9–7.69) [0.078]	6.41 (1.72–11.09) [0.007]	2.83 (0.99–8.17) [0.052]	5.86 (1.68–10.04) [0.006]
In the past month, total health expenditures						
Pre-frail	1.75 (1.12–2.75) [0.015]	−19.49 (− 47.34–8.36) [0.170]	1.82 (1.16–2.89) [0.010]	−17.31 (− 44.31–9.70) [0.209]	1.77 (1.11–2.8) [0.016]	−14.13 (− 39.51–11.25) [0.275]
Frail	2.66 (1.8–3.94) [< 0.001]	4.76 (− 24.47–33.99) [0.750]	2.92 (1.93–4.39) [< 0.001]	7.00 (− 22.03–36.03) [0.637]	3.03 (2.2–4.53) [< 0.001]	12.39 (− 16.32–41.10) [0.398]
	Model 4: adjusted		Model 5: adjusted		Model 6: adjusted	
	Logit OR (95% CI) [*p*-val.]	ME (95% CI) [*p*-val.]	Logit OR (95% CI) [*p*-val.]	ME (95% CI) [*p*-val.]	Logit OR (95% CI) [*p*-val.]	ME (95% CI) [*p*-val.]
In the past month, how much did you spend on consulting costs?						
Pre-frail	1.57 (0.93–2.64) [0.091]	−0.81 (− 9.02–7.41) [0.847]	1.6 (0.94–2.69) [0.080]	−0.52 (− 6.05–5.01) [0.854]	1.57 (0.92–2.69) [0.097]	1.03 (− 4.22–6.27) [0.701]
Frail	2.2 (1.4–3.49) [0.001]	3.82 (− 4.90–12.54) [0.390]	2.32 (1.45–3.67) [< 0.001]	5.27 (− 1.00–11.54) [0.099]	2.39 (1.46–3.9) [< 0.001]	5.84 (0.09–11.59) [0.047]
In the past month, how much did you spend on medications (drugs)?						
Pre-frail	1.54 (0.97–2.46) [0.069]	−1.01 (− 6.00–3.98) [0.692]	1.58 (0.99–2.53) [0.053]	−0.71 (− 5.75–4.33) [0.782]	1.6 (0.99–2.61) [0.055]	−0.95 (− 5.64–3.73) [0.690]
Frail	2.59 (1.72–3.86) [< 0.001]	8.37 (3.04–13.71) [0.002]	2.89 (1.9–4.35) [< 0.001]	9.96 (4.21–15.70) [0.001]	3.22 (2.08–5) [< 0.001]	8.61 (3.38–13.83) [0.001]
In the past month, how much did you spend on hospitalisation expenses?						
Pre-frail	1.63 (0.48–5.53) [0.437]	0.90 (−0.98–2.78) [0.349]	1.51 (0.44–5.1) [0.511]	0.66 (− 0.87–2.20) [0.396]	1.55 (0.45–5.37) [0.487]	1.13 (− 1.19–3.45) [0.339]
Frail	3.22 (1.12–9.3) [0.030]	5.94 (1.70–10.19) [0.006]	2.44 (0.84–7.1) [0.102]	5.14 (1.78–8.50) [0.003]	2.97 (0.99–9.03) [0.052]	10.43 (− 3.37–24.23) [0.139]
In the past month, total health expenditures						
Pre-frail	1.82 (1.16–2.89) [0.010]	− 12.27 (− 35.76–11.22) [0.306]	1.84 (1.16–2.89) [0.009]	−14.83 (− 40.04–10.38) [0.249]	1.93 (1.21–3.13) [0.006]	−7.932 (− 26.15–10.29) [0.394]
Frail	2.92 (1.95–4.35) [< 0.001]	13.29 (− 12.82–39.40) [0.319]	3.03 (2.01–4.57) [< 0.001]	10.70 (− 17.12–38.52) [0.451]	3.71 (2.39–5.75) [< 0.001]	17.35 (− 3.62–38.33) [0.105]

Reference group: robust (non-frail)

*ME* Marginal effect; *OR* Odd ratio; *CI* confident interval; *p-val*. *p*-value; ref.: reference category; Model 1: unadjusted; Model 2: adjusted by sex, age groups, and marital status; Model 3: adjusted by health insurance; Model 4: adjusted by area of residence and location; Model 5: adjusted by employment status and education level; Model 6: adjusted by sex, age groups, marital status, health insurance, area of residence, location, employment status and education level. The number of observations in all estimations is *n* = 860.

**Table 6:** Robustness check (estimation with different frailty thresholds)

	PR (95% CI) [*p*-val.]			
	Threshold at 0.25	Threshold at 0.31	Threshold at 0.35	Threshold at 0.41
Admitted to hospital overnight in last 12 months				
Pre-frail	4.8 (− 0.9–10.4) [0.191]	4.9 (− 0.9–10.7) [0.184]	4.9 (−0.8–10.7) [0.181]	5.3 (− 0.9–11.4) [0.172]
Frail	6.5 (− 1.2–14.1) [0.161]	6.7 (− 1.2–14.5) [0.159]	7.4 (− 1.4–16.2) [0.157]	7.4 (− 1.6–16.4) [0.164]
General practitioner in last 12 months				
Pre-frail	1.4 (0.9–1.9) [0.094]	1.5 (1.0–1.9) [0.062]	1.5 (1.0–2.0) [0.033]	1.6 (1.1–2.1) [0.017]
Frail	2.0 (1.4–2.7) [0.002]	2.1 (1.4–2.8) [0.001]	2.2 (1.5–2.9) [0.001]	2.2 (1.4–2.9) [0.002]
Consulted a specialist doctor in last 12 months				
Pre-frail	2.1 (0.7–3.5) [0.124]	2.2 (0.8–3.7) [0.094]	2.3 (0.9–3.8) [0.078]	2.3 (0.9–3.8) [0.076]
Frail	2.6 (0.9–4.2) [0.066]	2.4 (0.8–4.1) [0.078]	2.3 (0.7–3.8) [0.105]	2.3 (0.7–3.9) [0.118]
Self-medication in last 12 months				
Pre-frail	1.6 (1.1–2.5) [0.018]	1.6 (1.1–2.1) [0.012]	1.6 (1.2–2.1) [0.010]	1.7 (1.2–2.1) [0.006]
Frail	1.7 (1.2–2.2) [0.005]	1.7 (1.2–2.2) [0.006]	1.7 (1.2–2.3) [0.006]	1.6 (1.1–2.1) [0.020]
Traditional practitioner in last 12 months				
Pre-frail	1.8 (0.6–2.9) [0.197]	1.8 (0.6–2.9) [0.187]	2.0 (0.7–3.2) [0.125]	2.3 (0.9–3.6) [0.073]
Frail	3.2 (1.2–5.1) [0.032]	3.5 (1.3–5.7) [0.026]	3.7 (1.3–6.1) [0.025]	3.4 (1.2–5.7) [0.033]
	PR (95% CI) [*p*-val.]			
	Threshold at 0.25	Threshold at 0.31	Threshold at 0.35	Threshold at 0.41
Number of times being admitted to hospital overnight in last 12 months (with zeros)				
Pre-frail	2.8 (0.0–5.7) [0.201]	3.1 (0.0–6.2) [0.180]	3.0 (0.0–6.0) [0.184]	3.3 (0.1–6.6) [0.162]
Frail	5.4 (− 0.1–10.9) [0.119]	5.7 (− 0.1–11.6) [0.115]	6.6 (− 0.3–13.5) [0.113]	7.2 (− 0.5–14.8) [0.114]
Number of times consulting a general practitioner in last 12 months (with zeros)				
Pre-frail	1.8 (0.9–2.7) [0.093]	1.8 (0.9–2.7) [0.074]	1.9 (1.0–2.8) [0.059]	1.9 (1.0–2.8) [0.048]
Frail	2.2 (1.1–3.3) [0.029]	2.3 (1.1–3.4) [0.029]	2.3 (1.1–3.5) [0.030]	2.4 (1.1–3.7) [0.033]
Number of times consulting a specialist doctor in last 12 months (with zeros)				
Pre-frail	6.0 (0.8–11.2) [0.058]	6.0 (1.0–11.1) [0.052]	6.9 (1.1–12.6) [0.044]	6.7 (1.0–12.3) [0.049]
Frail	7.4 (0.7–14.1) [0.062]	7.6 (0.6–14.6) [0.066]	6.6 (0.2–12.9) [0.085]	7.6 (0.2–15.0) [0.082]
Cost variables with tobit model				
	PR (95% CI) [*p*-val.]			
	Threshold at 0.25	Threshold at 0.31	Threshold at 0.35	Threshold at 0.41
In the past month, how much did you spend on consulting costs?				
Pre-frail	2.9 (0.2–5.7) [0.039]	3.0 (0.3–5.7) [0.030]	3.3 (0.6–5.9) [0.015]	3.5 (1.0–6.1) [0.007]
Frail	5.2 (2.6–7.9) [< 0.001]	5.6 (2.9–8.4) [< 0.001]	6.0 (3.2–8.8) [< 0.001]	6.4 (3.5–9.4) [< 0.001]
In the past month, how much did you spend on medications (drugs)?				
Pre-frail	2.9 (0.6–5.3) [0.016]	3.2 (0.9–5.5) [0.007]	3.7 (1.5–6.0) [0.001]	3.9 (1.7–6.1) [0.001]
Frail	5.9 (3.6–8.2) [< 0.001]	6.2 (3.9–8.5) [< 0.001]	6.2 (3.7–8.6) [< 0.001]	6.8 (4.3–9.3) [< 0.001]
In the past month, how much did you spend on hospitalisation expenses?				
Pre-frail	4.7 (− 4.3–13.7) [0.306]	4.9 (− 3.8–13.6) [0.273]	4.3 (− 4.2–12.8) [0.322]	6.4 (− 1.9–14.7) [0.128]
Frail	8.6 (0.2–17.0) [0.044]	9.2 (0.6–17.7) [0.035]	11.1 (2.6–19.7) [0.011]	8.3 (− 1.2–17.9) [0.087]
In the past month, total health expenditures				
Pre-frail	3.7 (1.4–5.9) [0.001]	3.8 (1.6–6.0) [0.001]	4.2 (2.1–6.3) [< 0.001]	4.5 (2.3–6.6) [< 0.001]
Frail	6.1 (3.9–8.3) [< 0.001]	6.4 (4.2–8.6) [< 0.001]	6.5 (4.2–8.8) [< 0.001]	6.9 (4.5–9.3) [< 0.001]
Cost variables with two-part model				
	PR (95% CI) [*p*-val.]			
	Threshold at 0.25	Threshold at 0.31	Threshold at 0.35	Threshold at 0.41
In the past month, how much did you spend on consulting costs?				
Pre-frail	1.2 (− 3.4–5.8) [0.609]	2.7 (− 2.3–7.6) [0.288]	3.4 (− 1.2–8.5) [0.204]	3.6 (− 1.5–8.7) [0.163]
Frail	7.1 (0.8–13.3) [0.027]	6.5 (− 0.3–13.3) [0.061]	6.0 (− 1.4–13.5) [0.114]	6.3 (− 1.7–14.4) [0.124]
In the past month, how much did you spend on medications (drugs)?				
Pre-frail	−0.6 (− 4.9–3.7) [0.792]	0.1 (− 4.3–4.5) [0.955]	1.0 (− 3.3–5.3) [0.646]	3.0 (− 1.7–7.7) [0.208]
Frail	10.3 (4.9–15.7) [< 0.001]	11.5 (5.7–17.4) [< 0.001]	13.7 (7.1–20.3) [< 0.001]	13.2 (5.4–21.1) [0.001]
In the past month, how much did you spend on hospitalisation expenses?				
Pre-frail	4.2 (−3.3–11.7) [0.274]	4.0 (− 2.7–10.7) [0.243]	3.1 (− 1.8–8.0) [0.214]	6.5 (−3.3–16.3) [0.191]
Frail	8.7 (− 4.5–21.8) [0.197]	10.2 (− 5.7–26.1) [0.208]	13.6 (− 7.0–34.3) [0.196]	6.9 (− 6.3–20.2) [0.306]
In the past month, total health expenditures				
Pre-frail	−5.7 (−22.7–11.2) [0.510]	−2.9 (− 20.1–14.3) [0.743]	−0.7 (− 19.2–17.8) [0.939]	5.7 (− 15.0–26.4) [0.588]
Frail	21.9 (0.4–43.3) [0.046]	25.1 (1.7–48.5) [0.035]	28.4 (1.0–55.7) [0.042]	15.5 (− 12.4–43.5) [0.277]

When analyzing the effects of frailty on health expenditures using the two-part model, we find that being frail is associated with higher consulting expenditures, medication expenditures and hospitalization expenditures and that the magnitude of the effects are quite similar with that from the Tobit model. However, the effects of being pre-frail are not significant.

Both pre-frailty and frailty were associated with an increase in total health service expenditure for all models, as shown in Table 7. Under Model 6, the marginal effect (ME) for the frail sub-group was 6.1 (95% CI: 3.9–8.2, *p* < 0.001) and for the pre-frail group 2.8 (95% CI: 0.5–5.2, *p* = 0.020) in comparison with the non-frail sub-group for total health expenditure.

**Table 7:** Cross-sectional associations for frailty and health service expenditure

	Model 1: unadjusted ME (95% CI) [*p*-val.]	Model 2: adjusted ME (95% CI) [*p*-val.]	Model 3: adjusted ME (95% CI) [*p*-val.]	Model 4: adjusted ME (95% CI) [*p*-val.]	Model 5: adjusted ME (95% CI) [*p*-val.]	Model 6: adjusted ME (95% CI) [*p*-val.]
In the past month, how much did you spend on consulting costs?						
Pre-frail	2.7 (−0.4–5.7) [0.084]	2.9 (− 0.1–5.9) [0.058]	2.6 (− 0.4–5.6) [0.089]	2.6 (− 0.5–5.6) [0.096]	2.7 (−0.4–5.7) [0.084]	2.6 (− 0.3–5.6) [0.083]
Frail	4.8 (2.2–7.4) [< 0.001]	4.9 (2.4–7.5) [< 0.001]	5.3 (2.8–7.9) [< 0.001]	4.7 (2.1–7.3) [< 0.001]	5.0 (2.3–7.6) [< 0.001]	5.0 (2.4–7.6) [< 0.001]
In the past month, how much did you spend on medications (drugs)?						
Pre-frail	2.1 (−0.4–4.7) [0.102]	2.2 (− 0.3–4.8) [0.089]	2.0 (− 0.5–4.6) [0.117]	2.2 (− 0.4–4.7) [0.099]	2.3 (− 0.3–4.8) [0.082]	2.2 (− 0.3–4.7) [0.087]
Frail	4.9 (2.7–7.0) [< 0.001]	5.0 (2.8–7.2) [< 0.001]	5.3 (3.1–7.4) [< 0.001]	5.0 (2.8–7.2) [< 0.001]	5.5 (3.3–7.8) [< 0.001]	5.7 (3.5–8.0) [< 0.001]
In the past month, how much did you spend on hospitalisation expenses?						
Pre-frail	3.6 (−6.5–13.8) [0.480]	3.0 (− 6.8–12.9) [0.545]	3.8 (−6.4–13.9) [0.466]	4.1 (− 5.9–14.1) [0.425]	3.8 (−6.1–13.7) [0.456]	3.3 (− 6.4–13.0) [0.501]
Frail	8.9 (0.3–17.5) [0.044]	8.1 (− 0.4–16.5) [0.061]	9.3 (0.6–17.9) [0.036]	10.6 (2.1–19.1) [0.015]	7.9 (− 0.7–16.5) [0.071]	8.4 (0.2–16.7) [0.045]
In the past month, total health expenditures						
Pre-frail	2.7 (0.3–5.1) [0.028]	2.8 (0.4–5.2) [0.022]	2.6 (0.2–5.0) [0.031]	2.8 (0.4–5.2) [0.024]	2.8 (0.4–5.2) [0.021]	2.8 (0.5–5.2) [0.019]
Frail	5.1 (3.0–7.2) [< 0.001]	5.3 (3.2–7.4) [< 0.001]	5.5 (3.4–7.5) [< 0.001]	5.4 (3.3–7.5) [< 0.001]	5.6 (3.5–7.8) [< 0.001]	6.1 (3.9–8.2) [< 0.001]

Reference group: robust (non-frail)

*ME* Marginal effect; *CI* confident interval; *p-val*. *p*-value; ref.: reference category; Model 1: unadjusted; Model 2: adjusted by sex, age groups, and marital status; Model 3: adjusted by health insurance; Model 4: adjusted by area of residence and location; Model 5: adjusted by employment status and education level; Model 6: adjusted by sex, age groups, marital status, health insurance, area of residence, location, employment status and education level. The number of observations in all estimations is *n* = 860

When expenditure was disaggregated by provider, frailty was associated with increased expenditure across all models for aggregate (GPs and specialists) consulting costs (Model 6: ME =5.0, 95% CI: 2.4–7.6, *p* < 0.001) and medications (Model 6: ME =5.7, 95% CI: 3.5–8.0, *p =* < 0.001), while pre-frailty was not strongly associated with increased expenditure on either. With regard to hospitalisation expenses, all models showed increased expenditure with frailty with the exception of Models 2 and 5, while there was not strong evidence for an association of pre-frailty with increased hospitalisation expenditure under any model.

As described in the methodology section, we conducted a robustness analysis by varying the frailty threshold used. We find that increasing gradually the threshold from 0.21 to 0.41 did not lead to any significant change in the results reported for 0.21 as threshold for health service utilization and health service expenditure outcomes. As a result, our analysis is robust to the choice of the cut-points for classifying frailty.

## Discussion

In this population study of older Ivorians, we identified several key findings. Firstly, being frail was associated with increased health service utilisation across a range of services over the previous 12 months, with the exception of hospitalisations and specialist visits. Similarly, being frail increased total health expenditure across all services and all models, again with the exception of hospitalisation. On the whole, the finding that overall health service utilisation and expenditure tend to increase in association with frailty is consistent with previous work on this topic conducted [13–17, 28]. However, results for individual service types were mixed. In our sample, participants who were frail relied more heavily on primary care providers, traditional medical practitioners and on self-medication. The result for traditional and self-medication in particular was consistent with previous research on this topic within the Chinese context [27]. However, there was not an increased association with acute services such as hospitals and specialists, as has previously been observed in studies conducted within HICs [14, 17, 18, 29], even after controlling for a range of potentially confounding variables.

There are a number of potential reasons why frailty was not associated with an elevated tendency to be hospitalised in our study. Firstly, the health seeking experiences of participants in our sample are reflective of a widely recognised dichotomy between the biomedical model observed in Western medicine and the ethno-medical (socio-cultural) model practiced in many parts of the developing world [30]. As previous studies note, in such societies, traditional and cultural sources of health care may be preferred above, or used in tandem with, biomedical facilities, a trend which holds regardless of socio-economic circumstances [30–32]. Secondly, avoidance of hospital may be a response in contexts where in-hospital mortality for older adults is known, or perceived to be, high. Adebusoye and colleagues, commenting on the Nigerian experience, note that under these circumstances, older adults and their families may consider hospital as an absolute last resort [33]. Thirdly, cultural perceptions that medications provided within the acute setting are harmful may inhibit older adults from attendance [32]. Furthermore, and especially within rural areas, extended family members rather than formal health care providers are expected to provide the bulk of the care-giving responsibility to ailing relatives [30]. Lastly, it is likely that for those who are seeking more acute forms of medical care, access may be problematic [34], especially in more remote areas.

Aside from hospitalisations, our data did indicate that frailty was associated with increased health-seeking behaviour and increased use of health services, although this does not necessarily signify that the older people within our study were receiving appropriate and timely treatment for their condition. Even within HICs, where frailty research and awareness has been more extensive, frailty efforts remain focused on crisis management at the acute end of the spectrum, as opposed to preventative action within primary care settings [35]. As noted within one recent review, assessment and treatment of frailty within LMICs is further complicated by significant systemic, socio-cultural, geographical and workforce issues that will make service and policy responses to rapid population ageing challenging [36]. An important foundational step towards building future awareness and capacity for change will be the conduct of more research focused on older people’s frailty-related experiences and behaviour within LMIC settings [7, 36].

In our study, the proportion of our sample who were frail (60.0%) was relatively high compared with the few other studies that have been conducted to date in sub-Saharan Africa. A large Ghanaian sample with the same age range and based on a 40-item FI with a similar threshold (0.2) reported an age-standardised frailty prevalence rate of 37.9%. Other studies based in rural South Africa and Tanzania applied an alternative frailty definition (the Frailty Phenotype) and so were not directly comparable with our study [9, 37]. However, comparable rates have been observed in a remote Australian Aboriginal sample using a similar FI methodology and threshold [38]. More research is needed to explain such findings. As we anticipated, frailty was associated in our study with socio-economic measures such as being unemployed and having had limited educational opportunity, consistent with much prior research [39]. While we found that frailty was associated with rurality, other studies have found this not to be the case [18, 40]. This discrepancy is perhaps contingent on the local context of the study [18], including definitions of rurality.

Several strengths and limitations characterise this study. A key strength is that, to our knowledge, this is the first study conducted to date within SSA analysing frailty and its associations with health service utilisation and expenditure. As such, it expands the extant evidence base regarding the experience of frailty and ageing within LICs. A further strength is the inclusion of variables relating to usage of traditional medicine and self-medication in relation to frailty, again, the first to our knowledge to explore this relationship within SSA. One limitation of our study is that we were not able to determine the HIV status of participants. Given that sub-Saharan Africa has a large number of older adults living with HIV [41], the association with frailty might be expected to be a relevant factor. Further, although information on health care utilisation and expenditure was collected, accessibility to health services was not, a variable which may have influenced the association of these variables with frailty. Lastly, due to the cross-sectional nature of the data, causality could not be determined.

## Conclusion

Although frailty was associated with increased health service utilisation and expenditure among our older SSA sample, these trends were limited to primary and traditional forms of health care (GPs, self-medication, traditional practitioners) rather than more acute services (hospitals and specialists). It is currently unclear whether these trends reflect socio-cultural norms and preferences or a lack of access to acute services within the study setting. Further research is needed to ascertain the reasons behind frailty-related differential use of services among older Ivorians, taking into consideration also the potential role that HIV infection may play in influencing this relationship. Lastly, more research is needed on the cultural significance of frailty and its interface with health care systems, lest the assumption be made that the trends observed in HICs also apply elsewhere. Although the fact of ageing may be universal, it seems evident that the lived experience of frailty is not.

## Supplementary Information

**Additional file 1.**

## Data Availability

A sub-set of the data included within this study is available via Mendeley Data at this link: 10.17632/fhc7n2947t.1
