# Supplementary material for: Association of frailty with health service utilisation and health care expenditure in sub-Saharan Africa: evidence from Côte d’Ivoire
Source: BMC Geriatr. 2021 Jul 30;21:446. doi: 10.1186/s12877-021-02377-6 (PMC8323268; doi:10.1186/s12877-021-02377-6)
Supplement: Supplementary file 1 — Additional file 1. [file 12877_2021_2377_MOESM1_ESM.docx]

Supplement 1: Frailty Index Composition

|  | Question (French) | English translation | Response (Scoring) |
| --- | --- | --- | --- |
| 1 | Comment jugez-vous votre etat de sante physique en general? | How do you rate your state of physical health in general? | - (Bad) = 1 - (Intermediate) = 0.5 - All else = 0 |
| 2 | Votre etat de sante limite-t-il votre aptitude e faire vos activites? | Does your health status limit your ability to do your activities? | - (Severely) = 1 - (Just a little) = 0.5 - All else = 0 |
| 3 | Un medecin vous a-t-il diagnostique l'hypertension? | Has a doctor diagnosed you with hypertension? | - Oui (Yes) = 1 - Non (No) = 0 |
| 4 | Un medecin vous a-t-il diagnostique l'hypotension? | Has a doctor diagnosed you with hypotension? | - Oui (Yes) = 1 - Non (No) = 0 |
| 5 | Un medecin vous a-t-il diagnostique l'AVC? | Has a doctor diagnosed you with stroke? | - Oui (Yes) = 1 - Non (No) = 0 |
| 6 | Un medecin vous a-t-il diagnostique le diabete? | Has a doctor diagnosed you with diabetes? | - Oui (Yes) = 1 - Non (No) = 0 |
| 7 | Un medecin vous a-t-il diagnostique pour des problemes cardiaques? | Has a doctor diagnosed you with heart problems? | - Oui (Yes) = 1 - Non (No) = 0 |
| 8 | Un medecin vous a-t-il diagnostique une maladie pulmonaire chronique? | Has a doctor diagnosed you with chronic lung disease? | - Oui (Yes) = 1 - Non (No) = 0 |
| 9 | Un medecin vous a-t-il diagnostique l'Arthrite? | Has a doctor diagnosed you with arthritis? | - Oui (Yes) = 1 - Non (No) = 0 |
| 10 | Avez-vous des difficultes e marcher de longue distance (1 kilometre par exemple)? | Do you have difficulty walking long distance (1 kilometre for example)? | - Beaucoup (A lot) = 1 - Quelque peu (Somewhat) = 0.5 - All else = 0 |
| 11 | Avez-vous des difficultes e marcher de courte distance (100 metres par exemple)? | Do you have difficulty walking a short distance (100 meters for example)? | - Beaucoup (A lot) = 1 - Quelque peu (Somewhat) = 0.5 - All else = 0 |
| 12 | Avez-vous des difficultes e vous lever d’une chaise? | Do you have difficulty getting up from a chair? | - Beaucoup (A lot) = 1 - Quelque peu (Somewhat) = 0.5 - All else = 0 |
| 13 | Avez-vous des difficultes e monter plusieurs marches d’escalier simultanement? | Do you have difficulty climbing several steps at a time? | - Beaucoup (A lot) = 1 - Quelque peu (Somewhat) = 0.5 - All else = 0 |
| 14 | Avez-vous besoin d’assistance pour manger? | Do you need help to eat? | - Toujours (Always) = 1 - Souvent (Often) = 0.5 - All else = 0 |
| 15 | Avez-vous besoin d’assistance pour vous habiller? | Do you need assistance to dress? | - Toujours (Always) = 1 - Souvent (Often) = 0.5 - All else = 0 |
| 16 | Avez-vous besoin d’assistance pour vous laver? | Do you need assistance to wash? | - Toujours (Always) = 1 - Souvent (Often) = 0.5 - All else = 0 |
| 17 | Avez-vous besoin d’assistance pour utiliser les toilettes? | Do you need help using the toilet? | - Toujours (Always) = 1 - Souvent (Often) = 0.5 - All else = 0 |
| 18 | Avez-vous besoin d'assistance pour vous deplacer? | Do you need assistance to move? | - Toujours (Always) = 1 - Souvent (Often) = 0.5 - All else = 0 |
| 19 | Au cours de la semaine derniere, avez-vous eu des douleurs? | During the past week, did you have any pain? | - Beaucoup (A lot) = 1 - Quelque peu (Somewhat) = 0.5 - All else = 0 |
| 20 | Au cours de la semaine derniere, avez-vous eu besoin de vous reposer? | During the past week, did you need to rest? | - Beaucoup (A lot) = 1 - Quelque peu (Somewhat) = 0.5 - All else = 0 |
| 21 | Au cours de la semaine derniere, etes-vous senti faible? | During the past week, have you felt weak? | - Beaucoup (A lot) = 1 - Quelque peu (Somewhat) = 0.5 - All else = 0 |
| 22 | Au cours de la semaine derniere, avez-vous eu un manque d’appetit? | During the past week, did you have a lack of appetite? | - Beaucoup (A lot) = 1 - Quelque peu (Somewhat) = 0.5 - All else = 0 |
| 23 | Au cours de la semaine derniere, etiez-vous fatigue? | During the past week, were you tired? | - Beaucoup (A lot) = 1 - Quelque peu (Somewhat) = 0.5 - All else = 0 |
| 24 | Au cours de la semaine derniere, etes-vous senti tendu? | During the past week, have you felt tense? | - Beaucoup (A lot) = 1 - Quelque peu (Somewhat) = 0.5 - All else = 0 |
| 25 | Au cours de la semaine derniere, avez-vous eu peur? | During the past week, have you felt afraid/scared? | - Beaucoup (A lot) = 1 - Quelque peu (Somewhat) = 0.5 - All else = 0 |
| 26 | Au cours de la semaine derniere, etes-vous senti irritable? | During the past week, have you felt irritable? | - Beaucoup (A lot) = 1 - Quelque peu (Somewhat) = 0.5 - All else = 0 |
| 27 | Au cours de la semaine derniere, etes-vous senti depressif? | During the past week, have you felt depressed? | - Beaucoup (A lot) = 1 - Quelque peu (Somewhat) = 0.5 - All else = 0 |
| 28 | Au cours de la semaine derniere, avez-vous eu des difficultes à vous souvenir des choses ? | During the past week, did you have any difficulty remembering (with memory?) | - Beaucoup (A lot) = 1 - Quelque peu (Somewhat) = 0.5 - All else = 0 |
| 29 | Au cours de la semaine derniere, les douleurs ont-elles bouleversees vos activités ? | During the past week, did pain disrupt your activities? | - Beaucoup (A lot) = 1 - Quelque peu (Somewhat) = 0.5 - All else = 0 |
| 30 | Au cours de la semaine derniere, avez-vous eu des difficultes e vous concentrer ? | During the past week, did you have any difficulty concentrating? | - Beaucoup (A lot) = 1 - Quelque peu (Somewhat) = 0.5 - All else = 0 |
